# Factor associated with willingness to pay for prevention of cancer: a study of prostate cancer screening

**DOI:** 10.1186/s12962-023-00494-0

**Published:** 2023-11-21

**Authors:** Hiro Farabi, Najmeh Moradi, Aziz Ahmadzadeh, Seyed Mohammad Kazem Agamir, Abdolreza Mohammadi, Aziz Rezapour

**Affiliations:** 1 Barts and the London Pragmatic Clinical Trials Unit, Centre for Evaluation and Methods, Wolfson Institute of Population Health, Queen Mary University of London, London, UK; 2 Population Health Sciences Institute, Newcastle University, Newcastle upon Tyne, UK; 3 Insurance Research Center (IRC), Tehran, Iran; 4 Urology Research Center, Tehran University of Medical Sciences, Tehran, Iran; 5 Health Management and Economics Research Center, School of Health Management and Information Sciences, Iran University of Medical Sciences, Tehran, Iran

**Keywords:** Willingness to pay, Screening, Prostate cancer, Contingent valuation, Factor associated

## Abstract

**Introduction:**

This study investigates Iranian men’s willingness to pay (WTP) for prostate cancer (PCa) screening and influencing factor, along with the impact of information.

**Method:**

We assessed preferences for prostate cancer screening in 771 Iranian men aged 40 and above using an internet-based questionnaire survey. Participants received basic and complementary information, and their willingness to pay was determined through a payment card approach. A Wilcoxon test assessed the impact of information. We also analyzed prostate cancer screening demand and employed Heckman's two-step model to evaluate factors influencing the willingness to pay. Additionally, reasons for unwillingness to pay were explored.

**Results:**

Willingness to pay significantly decreased with complementary information relative to basic information (16.3$ vs 17.8$). Heckman model, using WTP based on basic information shows age, education, and monthly household expenditure positively influenced the decision to pay. In contrast, health status, expectations of remaining life and prostate problems history positively affect amount of WTP for PCa screening, and insurance coverage has a negative impact on it.

Majority of respondents (91%) supported PCa screening, with 82% expressing a willingness to pay. Common reasons for not paying include seeing screening as a public good (43%), financial constraints (35%), and having insurance (20%). The screening demand is price-sensitive.

**Conclusion:**

The basic mindset of Iranian men exaggerates the risk of prostate cancer. Reduced willingness to pay after receiving information reassures the reliability of their financial expectation. Taking into account the factors that influence PCa screening is essential for accurate planning and the successful implementation of this program.

## Introduction

Prostate cancer (PCa) ranked as the third most common cancer worldwide and the sixth most common among Iranian men [1, 2]. This form of cancer often referred to as an aging cancer [1, 3], typically remains silent and asymptomatic in its early stages [4]. However, it poses a considerable financial burden on the healthcare system [5]. In 2019, the financial burden of prostate cancer in Iran was estimated as 25.8 million US dollars [6]. With the 2020 incidence rate of prostate cancer (ASR, 21.1 per 100,000) and the latest population data for men aged 40 and above from the most recent census at the time of this research, it is approximated 23,000 men in Iran are at risk of developing prostate cancer.

Iran stands on the brink of a significant demographic shift, and rise of prostate cancer is anticipated. What distinguishes Iran from other nations is the rapid and intense nature of this shift. The Iranian population experienced substantial growth during the 1980s, and those born in that era are now in their forties. Consequently, this demographic transition is set to cause a noticeable change in Iran's population pyramid, leading it toward an aging demographic structure in the near future [7]. This underscores the critical importance of implementing preventive healthcare measures and increasing awareness to effectively combat the growing challenge of prostate cancer in Iran.

Prostate cancer treatment imposes a significant financial burden [6, 8] Early detection of cancer not only enhances the chances of successful treatment and prolonged life compared to diagnosis at later clinical stages [9, 10] but also play a cost reduction by mitigating the necessity for complex treatments associated with late-stage diagnoses [5, 11–13].

PCa screening plays a crucial role in improving cancer diagnosis rate, reducing mortality and enhancing the overall quality of life [14–16]. Despite its significance, mass screening for PCa has not been widely adopted in Iran. Implementing a cost-effective preventing program or mass screening is challenging.

Therefore, understanding the preferences of the participants and the extent of their financial involvement is essential, as it can help in formulating an optimal policy through their financial collaborations. Measuring the community’s willingness to pay provide a monetary value for the benefits of prostate cancer screening using prostate specific antigen (PSA).

Willingness to pay for health improvement represents the maximum amount an individual is willing to invest their health to achieve a better state [17, 18]. According to welfare economics theories an individual’s benefit from a service or intervention is determined by their maximum willingness to pay for that intervention or cost. Similarly, the benefit to society is measured by the total willingness of the population to pay for such services or interventions [17].

Various methods exist for assessing willingness to pay.The contingent valuation method is a widely accepted approach for valuing goods and services within the health sector [19]. With this method, individual do not make actual purchase. Instead, they are asked to imagine a hypothetical market for a product, placing themselves in a simulated situation and stating the highest price they would be willing to pay for that product [20]. This approach essentially measures willingness to pay by providing details and information about a non-market product within this hypothetical market setting [21].

Respondents may not have a complete understanding of a product's benefits and potential drawbacks, often overlooking possible scenarios when information is insufficient. As a result, their willingness to pay might be biased or inaccurate compared to situation with more comprehensive information about the product [22]. This variation can lead to less reliable assessments of people's willingness to pay [23, 24]. However, the influence of information on willingness to pay varies across studies. While some suggest that more information can decrease willingness to pay [25, 26], others have found that providing accurate information positively and significantly affects the acceptance and willingness to pay for goods [21, 27].

The extend to which a respondent values are closely tied to the impact of information [28]. For good significantly affecting personal utility, more information enhances understanding and willingness to pat. Conversely, for less personally connected goods, emotions play a significant role in willingness to pay. Therefore, assessing the impact of information on willingness to pay is essential for accurate valuation.

This study, used the contingent valuation method to assess men’s willingness to pay, examined the accuracy of stated willingness to pay, analyzed the impact of basic and complementary information on men's preferences, and investigated factors associated with prostate cancer screening willingness to pay among Irania men.

## Material and methods

### Subject

Iranian men aged 40 years and older comprised the target population for both the pilot and general phases of this study. Given the restrictions imposed by the covid-19 pandemic, we opted for an online questionnaire as our research tool. The questionnaire was hosted on an online platform^1^ and had certain restrictions such as not giving access to people who they express their age below 40. In the pilot stage, we randomly selected and surveyed a minimum of 40 men to complete the questionnaires. Based on the finding the pilot study, we estimated that a sample size of 845 would be adequate for the main research phase using the table provided by Michel and Carson [29] at a 90% confidence level.

All respondents provided responses after giving their informed written consent form. The questionnaires link was disseminated to men through social networks in July 2021. Respondents access the questionnaire by clicking on the provided link to view and complete it. To prevent duplicate responses, each IP address was permitted to complete the questionnaire only once. To ensure completeness and prevent missing data, answering to each question was linked to the next question. The questionnaire link was active for a duration of 14 days, and after obtaining a sufficient number of responses and reaching the desired sample size, the link was deactivated.

### Overview of the questionnaire

The questionnaire comprised six sections, developed based on data from a relevant systematic review [30] focused on men’s willingness to pay and associated factors and recommendations of an expert panel consisting of five specialist of urology and health economics. They include 1) Information and knowledge (which assessed each participant's awareness and knowledge about PCa and screening), (2) Attitudes (which examined their view on PCa, diagnosis and treatment method), (3) Experiences (which evaluated their past experiences and actions related to PCa, diagnosis, and treatment; (4) Demographic Characteristics; (5) Social Status; and 6) Health Status. These sections aimed to identify factors influencing WTP (refer to Table 1 for detail).

**Table 1:** Questionnaire variable description and interpretation

PSA history: “No history = 0, I have done = 1” to explore that respondent PSA history have an impact on the WTP and their payout level
Willingness to pay: “Do not want to pay = 0, Want to pay = 1” for extracting max of men WTP
Education: “Illiterate = 1, Elementary / Elementary end / Literacy = 2, First cycle / guidance / end of guidance / secondary = 3, Second cycle / secondary / intermediate 2 / no diploma = 4, Diploma / Pre- University = 5, Associate / Diploma = 6, Bachelor / Bachelor = 7, Master / Master / Professional Doctorate = 8, Specialized doctorate / postdoctoral = 9, Other = 10” to explore whether individual information (education) have an impact on the WTP and their payout level
Residency Area: “Village resident = 1, City resident = 2, Resident of the provincial capital = 3, Resident of the capital = 4” for evaluate whether the variable has an impact on the residents’ WTP and their payout level
Insurance: “Do not have = 0, have a basic insurance = 1, have a complementary insurance = 2, do not have complimentary insurance = 3″ f or detecting the effect of having or do not having insurance on men WTP and their payout level
Health Status: “low health Status = below 5, Intermediate health statutes = 5, adequate and high health status = between 5–10” to evaluate health Status' effect on men WTP and their payout level
Hospitalization: “No hospitalization history = 0, once = 1, Two and more = 2” to evaluate whether health status and hospitalization influences men WTP and their payout level
Prostate problem history: “No prostate problem history = 0, prostate problem history = 1” For evaluate respondent and his family prostate problem history effect on the men WTP and their payout level
Risk of incidence: “low risk = below 3, intermediate risk = 3, High risk = Further 3” for assessment of the effect of risk of PCa incidence rate respondent on the residents’ men WTP and their payout level
Age: “Year”, to evaluate whether respondent age affects the men WTP and their payout level
Expectation of remaining life: “Year” to evaluate whether respondent Expectation of remaining life on the men WTP and their payout level
Monthly Expenditure: “Dollar paid per Month” to evaluate whether respondent family expenditure effect on the men WTP and their payout level
Land lord status: “Proprietary = 1, Leased = 2, Other = 3” to evaluate land lording status effect on men WTP
Cancer History: “No cancer history = 0, Cancer history = 1” For evaluate respondent and his family any cancer history effect on the men WTP and their payout level
Occupational status: “Employed = 1, Unemployed (job seeker) = 2, Has an income without work = 3, Retirement = 4, Others = 5” to evaluate occupational status effect on men WTP
WTP: “do not willing to pay = 0, willing to pay = 1”

In addition, two types of information sheets namely basic and complementary information (see Box 1 and 3) were provided as well. These sheets contained objective facts as follow.

Box1: Basic information sheet.

**Table:** 

Prostate cancer is the second most common cancer in men, which increases with age. The risk of developing prostate cancer is very low at 40 years old and increases after 50 years old This cancer is asymptomatic in the early stages and its symptoms gradually appear with advancing the disease. It is not a fatal disease, but its progress reduces the patient's quality of life through difficult and expensive treatment methods. Prostate cancer screening measures prostate specific antigen (PSA) level in blood. Normally PSA is present in the blood of all men and its standard amount varies in different age groups If the PSA level in the blood is higher than the standard value for that age group, more examinations will recommend. These examinations are including Palpation of the prostate from the end of the large intestine, transrectal ultrasonography through anus and if necessary, prostate removing 6–12 samples of cells (biopsy). Complications of biopsy are rare and including bleeding and infection but detection rate is higher than 90%.	

The basic information card contained detail about “PCa, its characteristics in the early stages, the diagnostic method, and their potential complications. Respondents were presented with this card initially, followed by question about their maximum WTP. These questions were presented using a payment card (Box2), which depicted a hypothetical scenario including screening and various WTP options.

The WTPs options were determined using the mean and median values obtained in the pilot phase, taking into account the average cost PCa diagnosis and treatment at various stages of the disease. Additionally, a text box was provided as one of the payment card options for individuals who were willing to pay but preferred an amount below 1000 or above 10,000. This allowed participants to enter their specific WTP value for PCa screening.

Box2: Payment card.

**Table:** 

Suppose you are told that by paying a basic amount, you can benefit from early detection of prostate cancer by screening in just one next year. We want to know what is the maximum amount you are willing to pay? There is no right or wrong answer. The amount you say can be high or low. It's up to you. We want to know your opinion. (Amounts are based thousand Rail’s) 0 below-1000 1000 1250 1500 1750 2000 2250 2500 2750 3000 3500 4000 4500 5000 5500 6000 6500 7000 8000 10000 more than-10000	

Subsequently, participants received additional complementary information (Box3) which included details about limitations of the PSA test, potential false positive and negative results, supplementary diagnostic methods, and the complexity of treatment options at various stages of the disease. Following the provision of this information, respondents were asked to restate their maximum WTP. For those who indicated not willing to pay, additional inquiries were asked to understand the reasons behind their decision.

Box3: complementary information sheet.

**Table:** 

One of the limitations of a PSA is its lack of perfect accuracy. This means that every abnormal result does not mean that you have prostate cancer. Similarly, not every normal blood test result guarantee that a person is entirely healthy, and these individuals also need to repeat their annual checkups Out of every 100 individuals with an abnormal PSA who undergo biopsy, approximately 15 to 20 are diagnosed with prostate cancer. This likelihood increases with age In most cases, the cancer cells stay in place and do not spread In the early stages of prostate cancer, when cancer cells are confined with the prostate gland, treatment primarily involves regular follow-ups and medication. These are relatively simple and cost-effective methods, with a high probability of recovery often exceeding 95% If the cancer progresses and extends beyond the prostate gland but it is limited to the surrounding area, treatments such as hormone therapy, radiation therapy and partial prostatectomy (removing part of the prostate gland) may be recommended. These treatments are more expensive. Once cancer spreads to adjacent tissues and distant organs, traditional therapies become less effective and in such cases chemotherapy may be required to control spread of cancer cells. In advanced stages of prostate cancer, treatment options are less effective, more time consuming, costlier and associated with higher complications For example, prostatectomy surgery, may lead to issues such as impotence, impacting the patient's quality of life.	

The validity of the questionnaire was assessed using face validity. This process involved identifying and addressing any issues, ambiguities and shortcomings in the questionnaire that might have eluded the researcher’s initial review.

## Data analysis

After data collection phase, a thorough data set check and data cleaning process were carried out fixing or eliminate any incorrect, outlier, or unmatched data. The elicited WTP was then summarized and interpreted using both Mean and Trim Mean with the data categorized by different age group.

Due to the discrete nature of the selected values on the payment card, we used the minimal legal WTP (or ML-WTP) to determine the average WTP. According to this method, it is assumed that the true WTP falls between the selected value and the next higher one. Therefore, the ML-WTP was calculated by summing each amount on the payment card (Zi) multiplied by its relative frequency (Pi) as follow [31]:

$$ML-WTP= {\sum }_{i=0}^{N}{Z}_{i}{*P}_{i}$$

A Wilcoxon signed-rank test was employed to identify the differences between the means of the two sets of WTPs values gathered from the two types of information, both for all ages groups and for different age categories among the respondent men. This statistical test is utilized to assess the significance differences between two population means. It is conducted based on the discrepancies between two paired samples in the population, with the null hypothesis set to zero.

H_0_: ML- WTP With basic information = ML-WTP after complementary information

H_1_: ML- WTP with basic information ≠ ML-WTP after complementary information

The Wilcoxon test results are interpreted through the Z-score. A higher Z-score indicates a significant difference between the two sample, while and a smaller Z-score suggests greater similarity between them. The detailed results are presented in Table 4.

As the next step, we extracted the demand of prostate cancer screening. Given that one test per year is sufficient for monitoring individuals for PCa, there is no needs for anyone to purchase more than one unit. Consequently, an individual’s request for a PSA test is limited to a maximum of one, even if the price is zero. Consequently, the individual demand curve take the form of a step or bracket, where at prices less than or equal to each person’s WTP, the demand is equal to one unit, and at higher prices, individual demand becomes zero. Collective or demand or market demand is derived by horizontally summing all individual demands. Therefore, we calculated the collective demand function by assuming the demand for a PSA test item at prices equal to or less than each person’s WTP. The demand curve was redrawn and analyzed by removing 10% from the upper and lower values, both for the entire community and by different age groups.

The Heckman two-step model was employed to identify the factors influencing WTP among Iranian men. This method is a valuable approach for addressing provides a means of correcting for non-randomly selected samples. This model comprises two components: the Probit model which include factors affecting consumers 'decisions regarding WTP, and the linear regression model, which consists of independent variables influencing costumers actual WTP.

The Tobit Model within the Heckman Two-Step Model offer several advantages, including its efficacy in handling censored data, the capability to address selection bias, and its efficiency for mixed datasets. However, it relies on distribution assumptions and can be computationally intensive. Conversely, the Linear Model in the Heckman Two-Step Model is advantageous for its simplicity and computational efficiency, particularly with large datasets or when data is not censored. Nevertheless, it is not suitable for censored data and doesn't address selection bias. [32–34].

The linear regression component introduces a new variable known Inverse Mills Ratio (IMR), constructed using the estimated parameters from the Probit model. The overall significance of the model is determined and interpreted based on the IMR, which, if greater than critical level, indicates that factors influencing the participation are different from those affecting the WTP amount.

All statistical analyses were conducted using the Stata 17 and Excel software. To convert amount into US dollars, an exchange rate of 1 United Sates Dollar = 230,000 Iranian Rail used, based on the current exchange rate in the Iranian free market at the time of survey.

## Results

### Sample descriptive statistics

A total of 4446 individuals initially accessed the questionnaire, out of which 943 individuals were eligible and expressed their willingness to complete it. This initial response rate was 32% (943 out of 4446). Following data cleaning, 771 questionnaires were retained for analysis. Descriptive statistics presented in Tables 2 and 3 revealed that the knowledge level of Iranian men concerning the PCa and PSA testing is relatively low, with most respondents expressing a desire for more information on the subject. Furthermore, a small proportion of the respondents (11%) had undergone PSA testing, primarily for preventive and control purpose (45%), and some on the recommendation of a healthcare professional.

**Table 2:** Descriptive statistics of sample qualitative variables based on completed questionnaires (771 Obs.)

Variable name & notation	Variable selectable values	Frequency (qualitative variables)	
		Number	Percentage
PSA history	No history	183	31
	Have done	588	69
Willingness to pay	Do not want to pay	132	17
	Want to pay	639	83
Education	Illiterate	1	0
	Elementary/Elementary end/Literacy	3	0
	First cycle/guidance/end of guidance / secondary	17	2
	Second cycle/secondary/intermediate 2/no diploma	11	1
	Diploma/Pre-University	91	12
	Associate/Diploma	46	6
	Bachelor	268	35
	Master/Professional Doctorate	261	34
	Ph. D./Postdoctoral	65	9
	Other	1	0
Residency area	Village resident	26	3
	City resident	176	22
	Resident of the metropolitan area	290	37
	Resident of the capital	279	36
Insurance	No	74	10
	Have basic/national insurance	697	90
	Have complementary (Private) insurance	478	69
	Do not have complementary insurance	219	31
Health status	low health Status	20	3
	Intermediate health Status	34	4
	Adequate and high health Status	424	55
Hospitalization	No hospitalization history	332	43
	once	228	30
	Two or more	137	18
Prostate problem history	No prostate problem history	396	51
	Yes	375	49
Risk of incidence	low risk	322	42
	Moderate risk	369	48
	High risk	82	11
Residency status	Land lord	175	22
	Leased/ tenant	539	69
	Other	57	7
Cancer History	No cancer history	632	81
	Cancer history	139	18
Occupational status	Employed	41	5
	Unemployed (job seeker)	21	2
	Has an income without work	136	17
	Retired	541	70
	Other	32	4
WTP—basic information	Don’t willing to pay	132	9
	Willing to pay	639	81
WTP—complementary information	Don’t willing to pay	121	6
	Willing to pay	650	84

**Table 3:** Descriptive statistics of sample quantitative variables based on completed questionnaires (771 Obs)

Variable Name	Unit	Trim mean	Mean	S.D	Min	Max
Age	Year	–	46	10	26	80
Expectation of remaining life	Year	82.8	80	11.3721	0	100
Monthly Expenditure	US dollar	267	217	533	43	869

Table 2 indicates that 639 respondents (81%) who received the basic information card and 650 respondents (84%) who received the more complementary information card expressed a willing to pay for PCa screening. The average age of the respondents was 46 years, with an average monthly expenditure of 217 USD.

### WTP for PCa by age groups and different information

As per Table 4, the average WTP after receiving basic information was 17.83 USD, while it decreased to 16.36 USD after receiving complementary information.

**Table 4:** Sample data description of willingness to pay with basic info and complementary info and significancy of their difference (based on USD)

Age group	Info Type	Obs. No	Max	Mode	Average (ML-WTP)	Variance	SD	Z
All ages	WTP-basic info	771	4348	8.70	17.8	41,300	203	−8.7^***^
	WTP-complementary info	771	4348	8.70	16.3	33,493	183	
Age 40–49	WTP-basic info	513	4348	8.70	18.8	49,718	223	−7.2^***^
	WTP-complementary info	513	4348	8.70	18.5	46,913	217	
Age 50–54	WTP-basic info	105	870	8.70	17.9	8047	90	−2.5^**^
	WTP-Complementary info	105	130	8.70	11.6	422	21	
Age 55–59	WTP-basic info	85	870	8.70	18.8	10,179	101	−2.8^***^
	WTP-complementary info	85	435	8.70	14.4	2462	50	
Age 60–69	WTP-basic info	54	43	8.70	8.8	67	8	−2.4^**^
	WTP-complementary info	54	43	8.70	9.7	80	9	
Age 70 +	WTP-basic info	14	22	6.52	7.9	53	7	1.4
	WTP-complementary info	14	22	6.52	7.7	50	7	

^***^ Indicates P_value is less than < 0.01 (P-value < 0.01)

^**^ Indicates P-value is less than 0.05 (P-value < 0.05)

^*^ Indicates P-value is less than 0.1 (P-value < 0.1)

The results indicate that, in terms of age groups, the highest average WTP with both basic and complementary information was observed among men aged 40–49, and lowest average WTP was among men aged 70 and over.

### Iranian men’s demand for PCa screening

The frequency of cumulative market demand at various price levels provides insights into the number of people willing to undergo PCa screening at different price points.

Unfortunately, using the logarithm of WTP was not feasible to create a clear demand curve, especially since zero is one of the price axis values.

**Fig. 1:**
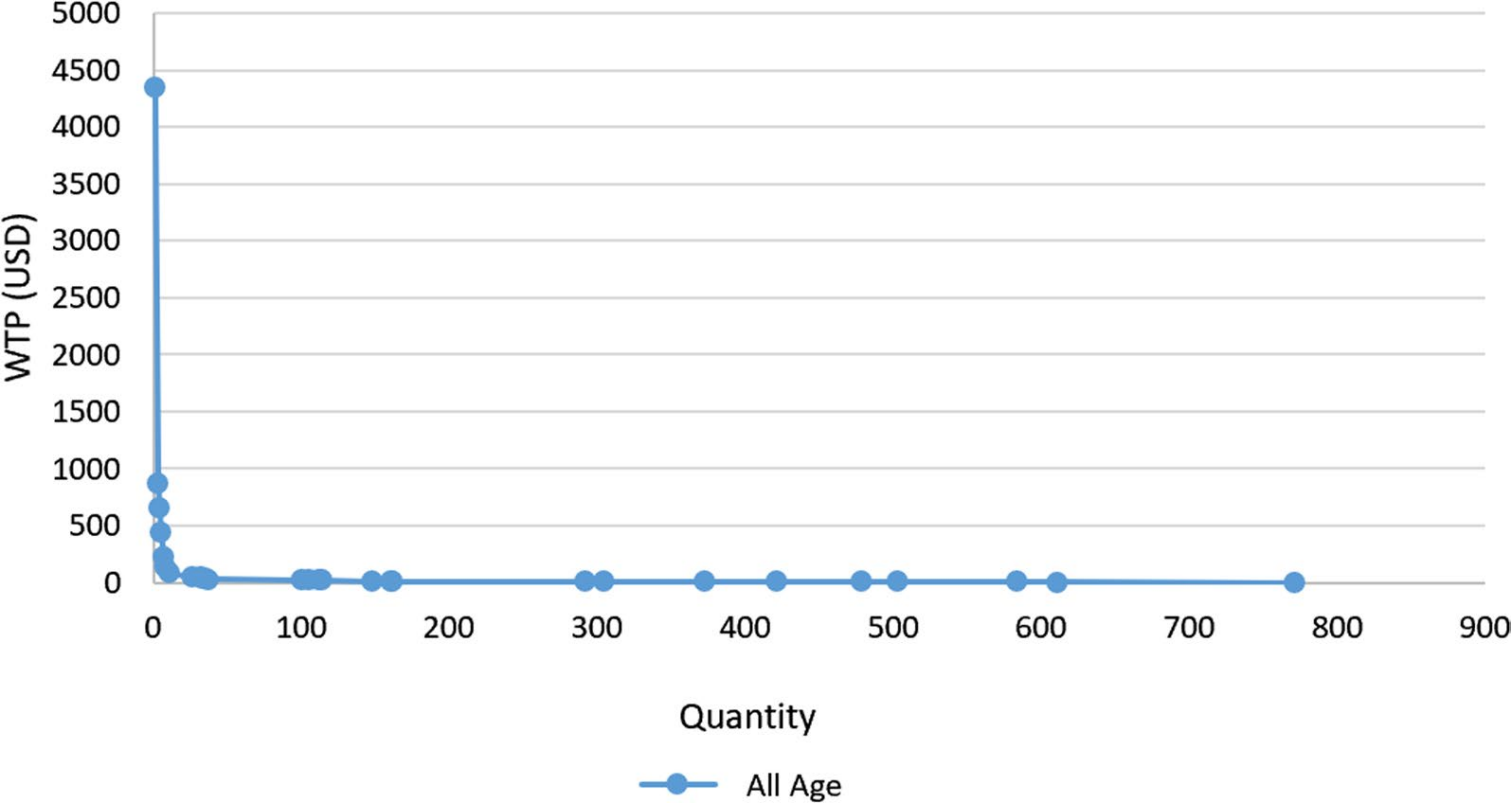
Men's cumulative demand for prostate cancer screening

Based on the graph (demand figures graphed using WTP basic information) in Fig. 1, the majority of men are unwilling to pay or pay less for this health service. The demand for PCa screening in price of 4348 USD and above is zero. However, as prices decrease to less than 22 USD, there is a slight upturn in demand. The demand curve exhibits a downward, hyperbolic shape, indicating that there are individuals who are willing to pay for this service even at very high prices.

**Fig. 2:**
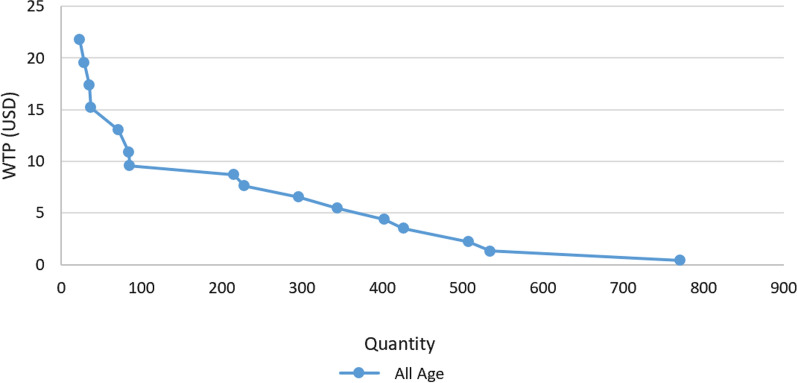
Men's cumulative demand for prostate cancer screening by eliminate 10% upper and lower values

Fig. 2 depicts the demand curve after eliminating 10% from the upper and lower values of the community. This graph continues to display a hyperbolic demand curve, indicating that as the price decreases, the slope of curve becomes less steep, signifying higher elasticity of demand. Notably, the primary demand falls within the price range of 0 USD and 22 USD. consequently, this price range can be considered as marketable price for PCa screening.

**Fig. 3:**
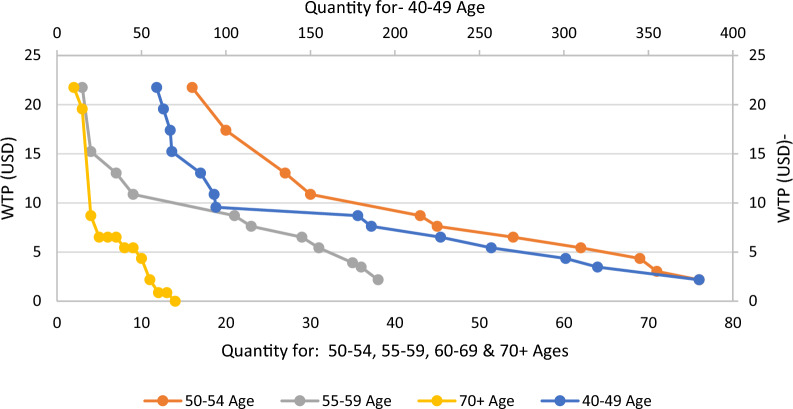
Decomposition of sample men's cumulative demand by age group classification

The decomposition of aggregate demand graphs by age groups as shown in Fig. 3, illustrates that nearly all demand curve are exhibit a decreasing and hyperbolic pattern. Furthermore, for all age groups, with the exception of those aged 70 years and above, the highest sensitivity (change in participation) concerning price is observed at willingness to pay values below 10 USD. However, in the age group of 70 years and above, this sensitivity occurs at 6 USD.

### The effect of information on men's WTP for PCa screening

The result of Wilcoxon test, as summarized in Table 4, indicates that providing additional information has a significant negative impact (with a statistic of -8.7 and p-value of zero) on the value of WTP among all men.

Furthermore, the Wilcoxon index estimated by different age groups reveals that the provision of more information results in a significant change in WTP for all age groups of men, except for those aged 70-year-old and above (with Wilcoxon’s Z value of 1.4).

### Model estimation and analysis of factors affecting the WTP

To extract these factors, we utilized the willingness to pay with basic information. The reason behind this choice is our belief that WTP under basic information represents a better representative of the population's preferences. This belief is further supported by the significant impact of information on WTP and it confirmed that respondents' behavior changes under the influence of the information in this regression. Heckman two-step model results are summarized in Table 5. To avoid severe collinearity, the use of high correlated variables in regression was avoided. For example, the variables of cancer history and history of prostate problems due to PCa involvement in both variables were highly correlated. Therefore, according to the research topic, the variable of history of prostate problems was included in the model. According to the results, the value of the Wald statistic was 22.35, and since this value is greater than the critical value of ${\chi }_{10, 95\%}^{2}=3.94$, the overall significance of the model is approved. Also, the inverse Mills ratio (IMR = -2.20) was significant at the level of 95% and shows that factors affecting the participation are not the same as factors that affect the WTP amount.

**Table 5:** Estimated Two-stage Hackman model—Determining the factors affecting the willingness of Iranian men to pay for PCa screening

Tobit Model			
Dep. Var.: willingness to pay	Coeff	S.E	Z
Education	0.07	0.039	1.76*
Residency area	−0.043	0.067	−0.063
Insurance	0.138	0.178	0.78
Hospitalization	0.042	0.073	0.58
Risk of incidence	0.078	0.052	1.49
Age	0.121	0.006	1.95*
Log (Monthly Expenditure)	0.508	0.028	1.79*
Land lord status	0.069	0.1	0.69
Cancer history	0.008	0.074	0.11
Occupational status	0.088	0.061	1.44
C	−1.04	0.524	−2**
Linear Model			
PSA history	−448,489	399,159.6	−1.12
Insurance	−2,615,089	727,058.6	−3.6***
Health Status	231,291.1	102,333.3	2.26**
Prostate problem history	669,531.1	347,948.7	1.96**
Expectation of remaining life	30,162.78	15,673.04	1.96**
C	−54,634.0	1,771,687	−0.03
Number of Obs	771		
Selected (No.)	640		
Non-Selected (No.)	131		
Wald Chi2	22.25		
Lambda /Mills (P-value)	−2.20 (0.028**)		

^***^ Indicates the P-value is less than 0.01 (P-value < 0.01)

^**^ Indicates the P-value is less than 0.05 (P-value < 0.05)

^*^ Indicates the P-value is less than 0.1 (P-value < 0.1)

Based on STATA output, in the first estimated in Table 5 reveals the factors influencing men's willingness to participate in payment. The finding indicate that age, education and monthly household expenditures had a positive and statistically significant impact at a 90% confidence level. This suggest that men with higher education and those with greater monthly living expenses are more likely to take part a screening program for early detection of PCa. Furthermore, age exhibited a positive and significant effect (at the 90% confidence level) on the willingness to engage in screening. The other variables integrated into the model, encompassing education level, and monthly household expenditure (utilized as an income proxy), also contributed to mens increased willingness to pay. The remaining variables in the model did not demonstrate a significant impact on participation.

In the second equation, the factors influencing the amount of WTP are presented. The results indicates that the insurance variable had a negative and significant effect at a 95% confidence level. This implies that men with insurance coverage tend to contribute less to screening compared to those without insurance coverage. Additionally, the health status of each individual exhibited a positive and significant effect on the WTP at the 90% confidence level. This suggests that men with a better health status are inclined to contribute more to screening costs. Furthermore, the variables related to the expectation of remaining life and a history of prostate problems also showed a positive and significant effect on the amount of WTP at the 90% confidence level. Therefore, men with a history of prostate problems and those with a longer expectation of remaining life expressed a greater willingness to pay.

## Discussion

Numerous studies have demonstrated that providing reliable information has a positive and significant impact on individuals’ willingness to pay. Therefore, in this study, our objective was to extract the preferences of Iranian men regarding PCa screening and assess the influence of information on their decision.

Despite the complete connection of the services examined in this study on the target community [28].providing information did not increase men's willingness to pay for them. The average WTP of all men with basic information was 17.8 USD, which decreased to 16.3 USD after providing the complementary information. The Wilcoxon test indicates that these differences are significant for all age groups except for those 70-year and above. This result aligns with previous studies, which have generally concluded that providing additional information does not lead to an increase in willingness to pay. For instance, Yasunga [35] found that men’s WTP did not decrease due to the provision of complementary information. Additionally, other studies [25, 26] presented participants with two different sets of information and observed that the information had a negative and significant effect on WTP, leading to decreased program valuation.

In Iranian society, many individuals with cancer are diagnosed in advanced stages, and leading to unfavorable outcomes. Consequently, when people become aware that their disease is PCa, one type of cancer, they tend to overestimate its risk. Therefore, providing additional information about this disease can help reduce their fear of it, especially in younger men, which may result in a decrease in their willingness to pay for screening. Naturally, older men tend to have more realistic perception of the disease from the outset. Therefore, the provision of additional information did not significantly decrease the willingness to pay for men in the highest age group.

The variance of willingness to pay with complementary information has decreased in comparison to WTP with basic information as age increases. This trend may be attributed to the income range of older men, which tends to be limited to retirement income. Consequently, men aged 60 years old and older are more homogenous in terms of willingness to pay.

Comparison of Iranian men's WTP with other studies reveals that Iranians men value the PCa screening more than Japanese (11 USD and 13 USD), as evidenced by studies conducted by Yasunga studies [26, 35]. However, Iranian men’s value it less than of the average WTP of European and American individuals [25, 36, 37]. This variations in willingness to pay can be attributed to cultural norms and economic factors. In Western societies, a strong emphasis on individualism and consumer culture leads to a higher willingness to spend. In contrast, Japan's collectivist culture, modesty, and conformity reduce personal spending. Iranian men, falling in between, are influenced by cultural values that prioritize individual well-being, while economic challenges, such as inflation, also play a role in shaping their spending habits. Indeed, while their inclination is generally towards personal welfare, economic challenges compel them to prioritize immediate savings over long-term health benefits. These differences illustrate the interplay of cultural norms and economic conditions in influencing individual behavior.

The highest willingness to pay was observed among men aged 40–49, with a decrease in willingness to pay as age increased. The most significant decline occurred at the age of 60. This can be attributed to the retirement age in Iran, which is typically 60 years old, leading to more limited expected income and reduced ability to pay. In contrast, men in the 40–49 age group are in their peak earning years and have greater capacity to pay for screening. WTP for PCa screening was not age-stratified in prior research. But in other type of cancers, diverse results emerged. For cervical cancer screening [38], younger individuals were less inclined to pay for early detection. However, in the case of breast cancer screening [39], younger respondents showed a stronger inclination to pay for early detection. These differences highlight the age-specific variations in willingness to pay for different types of cancer screenings.

Among those unwilling to pay, 43% considered PCa screening a public good and believed it should be provided by government. 35% cited financial constrains as their reason for non-payment, while 20% having insurance coverage and remaining participants express other reasons for not paying. According to participants expressions, they felt their monthly payments for primary health insurance and private medical insurance covered their health needs and additional payments for screening unnecessary.

Based on the sample demand graph for PCa screening (Fig. 1), most Iranian men tend to have screening for free or at low prices. This indicates a high sensitivity to price changes in the demand for PCa.This pattern is not uncommon and shares similarities with the demand for Colorectal cancer detection or screening [40]. While the cost of these tests isn't excessive, demand remains price-sensitive due to individual financial limitations and competing priorities [41].

The descriptive statistics of the completed questionnaires revealed that a significant proportion of respondents expressed a desire for more information. A minority of respondents reported prior experience with PSA testing primarily for prevention and control purposes, often following a physician’s recommendation. Providing complementary information to the public about PCa screening tests and their role in early diagnosis can encourage greater participation, even with associated cost. Such awareness might prompt many middle-aged and older individuals to proactively monitor their health status.

### Factors affecting the WTP

According to the results, age had a significant positive effect on WTP. This finding is consistent with the results of existing studies, so age is one of the main factors that will affect the men participant for paying or not.

The model shows that education is another factor associated with deciding for having pay or not for PCa screening. This finding is consistent with the results of Meyer and Newman studies [36, 37], which reported education as a positive factor in the WTP for PCa screening. Higher educational attainment appears to empower individuals with greater health knowledge and awareness [39], a relationship that is consistently supported by multiple studies [42, 43]. A further review of the responses of educated people revealed that men with higher education (in comparison with the average level of education in the community), not only had more information about the risk of disease, but also had a more accurate understanding of the benefits of early diagnosis and prevention. These men seem to pay more attention to their health and also do preventive actions due to their higher per capita reading. On the other hand, educated people usually have less uncertainty regarding their future expected income and hence, they can dedicate a budget to preventive actions easier than others. Their less uncertainty about income comes from high-paying jobs (and more savings and so, investment income) or at least a consistent regular income due to their specialization. Hence, these men are more likely to be willing to participate in paying for PCa screening.

Household expenditures were the third factor that is associated with deciding for having pay for PCa screening. People with higher monthly expenditures were more likely to decide to pay for PCa screening. Because people with lower spending levels face more severe budget constraints and are likely to have higher and more immediate priorities for allocating their limited income rather than to avoid potential future risks. In none of the existing studies household expenditures have been used to examine factors affecting WTP, but literature confirmed that low income and less wealthy individual demonstrate less WTP [44]. The reason for using expenditure instead of income in the present study is that the tendency to self-declaration income is less and the figures related to expenditure are more accurate and the results are more reliable. Therefore, considering that income and expenditures have a high correlation, household expenditures are the best proxy for measuring income. In the reviewed studies [25, 26, 35–37, 45] income has been reported as a factor that has a positive effect on WTP. By using expenditures as a proxy for income, it can be said that our result was consistent with the results of global studies.

Other variables, including employment status, residence (urban or rural), residency status, family history of cancer, insurance coverage, hospitalization history and the risk of PCa incidence did not have a significant effect on deciding for pay for PCa screening. Our finding contrast with those of previous studies, indicated a positive and significant impact for being at risk of PCa [37]*,* having family history of PCa and history of hospitalization [26, 35].

### Factors affecting the level of WTP

Men's health status (measured as respondents' self-expression about their health) had a positive and significant effect on the amount of WTP. As Individual are willing to pay for services to avoid the negative health risks [46], those with better health tended to pay more for PCa screening. It can be interpreted as the high valuation of complete healthiness in their preferences; because when they are not healthy or not feeling well, they think taking preventive action cannot be so helpful to recover their overall well-being and so, they have less tendency to pay. For this reason, by paying more money, the healthier men were trying more to avoid the risk of PCa and somehow invest in their future health by taking preventive proceedings and early diagnosis. This factor was not significant in previous studies.

The presence of a family history of prostate problems significantly influenced men's WTP level, with those having at least one male family member who had experienced a prostate problem showing a higher willingness to pay. Indeed, having a family member with a negative experience increases the WTP amount to prevent adverse effects [47].This observation suggests that individuals who have firsthand experience with the physical, mental, and financial challenges posed by prostate problems are more likely to invest in PCa screening and early detection. Such individuals may also adopt a more proactive approach to prevent the recurrence of prostate issues. This finding aligns with the results of other studies [25, 35, 36]that have also reported a positive impact of a family history of prostate problems on the amount of WTP. Notably, this positive relationship between family history and WTP has been observed in other types of cancers, such as breast and ovarian cancers as well [48].

The third factor that significantly positively influenced the amount of willingness to pay was expectation of remaining life (in this study this variable was obtained by answering the question how long they expected to live). The positive effect of expectation of remaining life on the amount of WTP indicates that individuals who are more optimistic about their lifetime are willing to pay more for early PCa diagnose through screening. One possible reason for this positive effect is that individuals who expect a longer remaining life anticipate a more extended period of potential suffering if they were to become ill. As a result, they are more inclined to invest in the benefits of good health rather than enduring the consequences of illness for the rest of their lives. From another perspective, the willingness to pay for healthcare services benefits individuals by extending their lifespan [49]. Therefore, those in this study who anticipate living longer believe that by paying for early prostate cancer screening, they are aligning with their expectations. This variable has not been examined in previous studies.

The presence of insurance coverage had a significant negative effect on the WTP amount. This negative relationship is likely because respondents considered out-of-pocket when expressing their WTP. They expected, similar to other health care services, that a substantial portion of screening cost would be covered by their insurers. On the other hand, insurance coverage, by making early detection more affordable [39] can lead to a decreased willingness to pay for it. The findings of this study are in alignment with a prior investigation concerning prostate cancer [25]. However, research in distinct domains, such as breast cancer screening [39] and genetic testing for cancer risk [50], reveals a inconsistence result where having insurance coverage exerts a positive significant on individuals' willingness to pay (WTP). This discrepancy underscores the necessity to recognize the diverse impacts of insurance coverage on various healthcare services.

In contrast, the history of PSA testing had no significant effect on the WTP amount in this study. While, Yasunga and Pedersen studies [25, 26]reported positive and negative effects on the effect of PSA test history on WTP, respectively. The results of us diverge significantly from both of these studies, and given the inconsistency of the findings, it is challenging to provide a conclusive analysis or generalize the effect of PSA history on WTP for PCa screening.

While our study provides valuable insights into factors influencing the willingness to pay for PCa screening among Iranian men, it's important to acknowledge that some relevant aspects remain unexplored. We recommend these avenues for future research, which could further enhance our understanding of this topic.

- In particular, future research endeavors can prioritize the enhancement of awareness and willingness to pay for prostate cancer screening, with special attention to individuals who may initially lack interest in the screening process.
- Investigating the effectiveness of diverse awareness methods, encompassing public health campaigns, community outreach, online resources, educational materials, and the support provided by advocacy groups, is of paramount importance in shaping men's willingness to pay.
- Moreover, collaborating with healthcare providers to tailor interventions targeting specific age groups and risk profiles is essential to maximize the impact of screening initiatives.
- Beyond these considerations, future studies can delve into additional factors that may have been overlooked, including the role of social support systems and the influence of specific insurance packages, all of which are integral in bolstering mass screening efforts for prostate cancer.

These comprehensive investigations can pave the way for more effective and inclusive prostate cancer screening programs.

## Conclusion

The basic mentality of Iranian men considers the risk of prostate cancer more dangerous than reality. The less WTP due to information provided assures us that the expectation from men's financial participation is reliable and trustworthy. The comprehensive analysis of the various factors that shape prostate cancer screening is of paramount importance to ensure that the program is meticulously designed to cater to the specific needs and dynamics of the population it serves. By taking into account elements such as health status, personal expectations, and family history, program planners can create a more tailored and effective strategy. This strategic approach not only fosters accurate planning but also enhances the likelihood of the program's overall success in promoting men's health and cancer prevention.

## Limitations

- We aimed to address the challenges of collecting data from the entire community, considering the considerable time and financial resources required, by utilizing sampling. However, we acknowledge that our sample size, though obtained through a relevant contingent evaluation method, represents only approximately 0.007% of the entire Iranian male population aged 40 years and above, potentially limiting the generalizability of our findings to the broader population. While our goal was to secure a diverse and representative sample, but constraints in resources and the disruptions caused by the COVID-19 pandemic made it impractical to obtain a larger sample.
- The use of social networks for information compilation has inherent challenges, including potential bias, variable data quality, privacy concerns, and complex data ownership issues. To address these concerns, we've taken measures to mitigate bias, verify data quality, protect privacy, and obtain permissions while adhering to ethical standards. The benefits of accessing unique datasets through social networks outweigh these potential problems, and we've made every effort to ensure the reliability and validity of the data.
- In assessing male participation rate, it was observed that the response rate was low (32%) and the study may have a self-selective bias.
- In addition, using a web-based questionnaire and releasing survey links via social networks come with limitations, including self-selection bias, limited control over the sample, and challenges in verifying respondent authenticity. This method may attract participants who are more active on social media or have specific interests, potentially leading to a non-representative sample. Additionally, ensuring data quality and preventing multiple responses from the same individual can be challenging.
- Due to Covid-19 restrictions, the questionnaire was made available to participants online and only men who were willing and able to participate completed the questionnaires. Therefore, the collected questionnaires did not include the information from those men who are concerned about their health but did not want to complete the questionnaire and the sample used to extract the benefit may be slightly biased towards the whole community.
- The sample size of participants who completed the WTP questionnaire in the age group of 70 years and above was smaller than the other groups. It seems that one of the main reasons was the Covid-19 pandemic and the impossibility of face-to-face interviews with the participants. Since the questionnaires were provided to the respondents on the Internet and may be due to reasons such as unwillingness to use social media or lack of sufficient knowledge in using this space or smartphones, they participate less in this study.
- The payment card method in contingent valuation, while advantageous in some respects, has its limitations. One significant limitation is the potential for starting point bias, wherein the sequence of payment amounts presented on the card may influence respondents' willingness to pay. To mitigate this bias, we designed our payment card survey to randomize the presentation order of payment amounts to each respondent. Moreover, the payment card method is susceptible to scenario bias, as the provided scenarios might not fully encompass the intricacies of real-life choices and trade-offs, potentially yielding different results compared to other valuation methods. To mitigate this, we aimed to craft scenarios that closely resembled real-world choices and trade-offs, thus reducing scenario bias. Additionally, the quality and accuracy of data collected through the payment card method may be influenced by factors such as the clarity and design of the payment card and respondents' understanding of the task, introducing response variability. To address this concern, we conducted a pilot test to ensure the clarity and effectiveness of the payment card and the respondents' understanding of the task.

## Data Availability

The datasets used and/or analyzed during the current study are available from the corresponding author on reasonable request.

## Abbreviations

WTP: Willingness to Pay
CVM: Contingent Valuation Method
PCa: Prostate Cancer

## Footnotes

1 PorsLine (https://porsline.ir) is an online platform for designing and sharing questionnaire of surveys in Iran.
